# Trimethylamine-N-Oxide Levels Are Similar in Asymptomatic vs. Symptomatic Cerebrovascular Atherosclerosis

**DOI:** 10.3389/fneur.2021.617944

**Published:** 2021-03-19

**Authors:** Miriam Heyse, Christine Schneider, Peter Monostori, Kathrin V. Schwarz, Jana Hauke, Katharina Drüschler, Anne Berberich, Markus Zorn, Peter A. Ringleb, Jürgen G. Okun, Sibu Mundiyanapurath

**Affiliations:** ^1^Department of Neurology, Heidelberg University Hospital, Heidelberg, Germany; ^2^Metabolic and Newborn Screening Laboratory, Department of Pediatrics, University of Szeged, Szeged, Hungary; ^3^Division of Child Neurology and Metabolic Medicine, Dietmar-Hopp Metabolic Center, Center for Child and Adolescent Medicine, University Hospital Heidelberg, Heidelberg, Germany; ^4^Central Laboratory, Department of Internal Medicine, University Hospital Heidelberg, Heidelberg, Germany

**Keywords:** cerebrovascular, atherosclerosis, gut microbiome, ischemic stroke, carotid artery stenosis

## Abstract

**Introduction:** Trimethylamine-N-oxide (TMAO) is correlated with atherosclerosis and vascular diseases such as coronary heart disease and ischemic stroke. The aim of the study was to investigate whether TMAO levels are different in symptomatic vs. asymptomatic cerebrovascular atherosclerosis.

**Methods:** This was a prospective, case–control study, conducted at a tertiary care university hospital. Patients were included if they had large-artery atherosclerosis (TOAST criteria). Symptomatic patients with ischemic stroke were compared with asymptomatic patients. As primary endpoint, TMAO levels on admission were compared between symptomatic and asymptomatic patients. Univariable analysis was performed using Mann–Whitney *U* test and multivariable analysis using binary logistic regression. TMAO values were adjusted for glomerular filtration rate (GFR), age, and smoking.

**Results:** Between 2018 and 2020, 82 symptomatic and asymptomatic patients were recruited. Median age was 70 years; 65% were male. Comparing symptomatic (*n* = 42) and asymptomatic (*n* = 40) patients, no significant differences were found in univariable analysis in TMAO [3.96 (IQR 2.30–6.73) vs. 5.36 (3.59–8.68) μmol/L; *p* = 0.055], GFR [87 (72–97) vs. 82 (71–90) ml/min^*^1.73 m^2^; *p* = 0.189] and age [71 (60–79) vs. 69 (67–75) years; *p* = 0.756]. In multivariable analysis, TMAO was not a predictor of symptomatic cerebrovascular disease after adjusting for age and GFR [OR 1.003 (95% CI: 0.941–1.070); *p* = 0.920]. In a sensitivity analysis, we only analyzed patients with symptomatic stenosis and excluded patients with occlusion of brain-supplying arteries. Again, TMAO was not a significant predictor of symptomatic stenosis [OR 1.039 (0.965–1.120), *p* = 0.311].

**Conclusion:** TMAO levels could not be used to differentiate between symptomatic and asymptomatic cerebrovascular disease in our study.

## Introduction

Trimethylamine-N-oxide (TMAO) has been proposed as a biomarker for the gut microbiome (1). Its precursor, Trimethylamine (TMA), is produced by the gut microbiome after consumption of L-carnitine, betaine, and choline (for example, found in egg yolk, leguminous plants, fish, and red meat) and is then oxidized to TMAO in the liver and secreted via urine afterwards (1). TMAO has been shown to be elevated in patients with coronary heart disease and ischemic stroke (2, 3). It has also been shown to predict major adverse cardiovascular events (2). Furthermore, ischemic stroke as a complication of carotid artery stenting (CAS) was predicted by TMAO levels (4). In this study, 268 patients who underwent CAS had a MRI within 3 days after the procedure. TMAO levels were measured within 3 days before the procedure. Patients with new diffusion-weighted imaging (DWI) lesions had significantly higher TMAO levels compared to patients who did not show new DWI lesions. Patients with new DWI lesions were also more likely to have symptomatic carotid artery stenosis (75 vs. 60%, *p* = 0.01). On the other hand, 25% of patients with asymptomatic carotid artery stenosis also had new DWI lesions. The results raised the question whether TMAO levels are higher in symptomatic vs. asymptomatic cerebrovascular atherosclerosis and, if yes, might be used to predict progression of asymptomatic cerebrovascular atherosclerosis or imminent stroke. We therefore set out to compare TMAO levels in symptomatic and asymptomatic patients with ischemic stroke due to large-artery atherosclerosis.

## Methods

### Patients

This study was a prospective, single-center, case–control study, including patients with symptomatic and asymptomatic large-artery atherosclerosis between March 2018 and July 2020 at a tertiary university hospital. Symptomatic patients were included if they had an ischemic stroke (diagnosed clinically or using imaging by an experienced vascular neurologist) within the last 24 h and were submitted to our stroke unit. They had to be older than 18 years and the etiology of the stroke had to be large-artery atherosclerosis according to the TOAST criteria [Trial of Org 10172 in Acute Stroke Treatment (5), defined by an experienced vascular neurologist]. Asymptomatic patients were recruited as outpatients in our neurovascular clinic. They had to be older than 18 years and suffer from asymptomatic large-artery atherosclerosis according to TOAST criteria. Patients were considered asymptomatic if they did not have an ischemic stroke or TIA within the last 6 months, in accordance with a randomized trial on asymptomatic carotid artery stenosis (6). Besides TMAO and glomerular filtration rate (GFR), age, cardiovascular risk factors (hypertension, diabetes, atrial fibrillation, current smoker, coronary heart disease, and peripheral artery disease) and degree of stenosis were recorded.

Exclusion criteria were current chemotherapy, severe anemia, antibiotic therapy within the last 30 days, patients receiving pre- or probiotics, and history of trimethylaminuria.

Some results of patients in this study have already been published as part of a study on TMAO levels in patients with and without ischemic stroke (7).

### Blood Sampling and Analysis

Plasma samples of symptomatic patients were collected immediately on admission to the emergency room. Samples were then stored at −20°C until analysis. Plasma of asymptomatic patients was drawn at any time during their visit in the neurovascular clinic. Preparation of the samples was performed as previously described (8). Briefly, samples (10 μl) were pipetted into centrifuge tubes (1.5 ml). Then, a mixture of methanol (MeOH) and acetonitrile (ACN) with a ratio of MeOH:ACN of 25:75, v/v, at 4°C and 50 μl of a 2 μmol/l d9-Trimethylamine-N-oxide (Cambridge Isotope Laboratories) solution in MeOH were added. The mixture was mixed for 30 s followed by 10-min incubation and centrifugation at 18,000 × gmax for 5 min. The supernatant (150 μl) was transferred to a cavity of a 96-well microplate. Quantification was performed with an external nine-point calibration using the peak area ratio of TMAO relative to its internal standard d9-TMAO. For calibrators, fetal bovine serum (FBS) was used as matrix. After spiking with TMAO in the range of 0–200 μmol/l, calibrators were prepared as described above. Quality controls were included in each experimental series (3, 15, and 75 μmol/L in FBS). The microplate was sealed with a preslit adhesive foil after transfer of all calibrators, quality controls, and samples.

Liquid chromatography–tandem mass spectrometry (LC-MS/MS) analyses were performed using a Waters XEVO TQS system (Waters, Eschborn, Germany) equipped with an electrospray ion source. The instrument was controlled with MassLynx 4.1 software. For chromatographic separation, a HILIC column (Waters Acquity UPLC BEH Amide 100 × 2.1 mm; 1.7 μm) with a corresponding pre column (Waters Acquity UPLC BEH Amide VanGuard, 5 × 2.1 mm; 1.7 μm) was used in isocratic mode. During a 3-min chromatographic run, eluent A [10 mmol/L ammonium formate in ultrapure water (H_2_OmQ) and ACN (H_2_OmQ:ACN 95:5, v/v)] and eluent B (ACN) were applied with a mixing ratio of 42% A and 58% B at a flow rate of 0.4 ml/min. The injection volume was 1 μl. Both TMAO and its corresponding standard were analyzed using a multiple reaction monitoring (MRM) experiment containing their most abundant mass transitions: (TMAO: 76.1 Da 59.1 Da; d9-TMAO: 85.1 Da 68.1 Da; cone voltage: 40 V; collision energy: 11 V) in positive ion mode at a flow rate of 0.4 ml/min for 3 min.

Our central laboratory calculated GFR using the measurement of serum creatinine according to the chronic kidney disease epidemiology collaboration equation (CKD-EPI). All values of GFR are reported in ml/min^*^1.73 m^2^.

### Doppler Sonography

The degree of the artery stenosis was determined as previously described (9). Briefly, continuous-wave Doppler sonography (4 MHz extracranial Doppler probe, SONARA system, medilab®, Estenfeld, Germany) and a linear 5- to 10-MHz duplex probe were used to define the degree of stenosis according to the NASCET criteria defined by the German Society of Sonography in Medicine (DEGUM) (10). Transcranial Doppler sonography was performed with a 2-MHz pulse-wave probe using the SONARA system.

### Statistical Analysis

Descriptive analysis was performed using absolute numbers and relative rates for categorical variables; median and interquartile range (IQR) were used for continuous variables. Correlations were calculated using univariable Spearman-Rho coefficients. Further univariable comparisons were performed using Mann–Whitney *U* test or Kruskal–Wallis test for continuous variables and chi-square test or Fisher's exact test for categorical variables. Fisher's exact test was used if the number of participants in one field was below 5. Binary logistic regression was used for multivariable analysis, symptomatic vs. asymptomatic large-artery atherosclerosis being the dependent variable while age, smoking, and GFR were the independent variables. The independent variables were chosen if there were known correlations with TMAO or if they were significantly different in univariable analysis. We reported odds ratios (OR) with 95% confidence interval (CI). Alpha level was set to 0.05, SPSS version 24 was used to carry out the analyses. All reported *p*-values are two-sided. Pretesting for type of distribution was avoided to rule out error accumulation (11).

### Ethical Approval and Patient Consent

The study has been approved by the local ethics committee (S-521/2017). Written informed consent was obtained by all participants or their legal representative before enrollment.

## Results

In total, 82 participants were included in the study between 2018 and 2020. Forty-two suffered an ischemic stroke and 40 (49%) participants showed asymptomatic cerebrovascular atherosclerosis. Forty-one symptomatic patients were included from a previously published study (7). One symptomatic patient was included while screening for asymptomatic patients. Median age of the included patients was 82 years (IQR: 62–77). Symptomatic patients were mostly male [53 participants (65%)]. Most of the patients suffered from hypertension [77 (94%)], while only few participants suffered from atrial fibrillation [7 (9%)]. More symptomatic patients were current smokers compared to asymptomatic patients [18 (43%) vs. 8 (20%); *p* = 0.026]. Blood sampling in symptomatic patients was performed in a median of 585 (275–1,043) min in symptomatic patients. Asymptomatic patients' plasma was analyzed at any time during their outpatient appointment. More details on baseline parameters can be found in Table 1.

**Table 1:** Baseline characteristics and TMAO values.

**Parameter *n* (%) or median (IQR)**	**Total**	**Symptomatic**	**Asymptomatic**	***p*-value**
Total number of patients	82	42	40	–
Age	70 (62–77)	71 (60–79)	69 (67–75)	0.507
Male gender	53 (65%)	28 (67%)	25 (63%)	0.693
Atrial fibrillation	7 (9%)	3 (7%)	4 (10%)	0.643
Hypertension	77 (94%)	40 (95%)	37 (93%)	0.672
Diabetes	23 (28%)	14 (33%)	9 (23%)	0.275
Coronary heart disease	18 (22%)	11 (26%)	7 (18%)	0.342
Peripheral artery disease	9 (11%)	3 (7%)	6 (15%)	0.307
Current smoker	26 (32%)	18 (43%)	8 (20%)	0.026
GFR (ml/min*1.73 m^2^)	83 (71–94)	87 (72–97)	82 (71–90)	*p* = 0.189
TMAO (μmol/L)	4.41 (2.72–8.02)	3.96 (2.30–6.73)	5.36 (3.59–8.68)	*p* = 0.055
Platelet inhibition	47 (57%)	15 (36%)	32 (80%)	*p* < 0.001
Statin	44 (54%)	11 (26%)	33 (83%)	*p* < 0.001
Antihypertensive medication	64 (78%)	29 (69%)	35 (88%)	*p* = 0.044
Anticoagulation	7 (9%)	2 (5%)	5 (13%)	*p* = 0.210

*GFR, glomerular filtration rate; IQR, interquartile range*.

Most of the patients suffered from internal carotid artery stenosis [57 (70%)]. The rate of carotid artery stenosis was lower in symptomatic compared to asymptomatic patients {25 [60% vs. 32 (80%)]}. Only symptomatic patients suffered from occlusions of brain-supplying arteries (Table 2).

**Table 2:** Location and degree of stenosis or occlusion.

**Location**	**Degree**	**Total** **(*n* = 82)**	**Symptomatic (*n* = 42)**	**Asymptomatic (*n* = 40)**	***p*-value**
ICA	50%	15 (18%)	3 (7%)	12 (30%)	0.029
	70%	14 (17%)	7 (17%)	7 (18%)	0.920
	80%	10 (12%)	2 (5%)	8 (20%)	0.035
	90%	13 (16%)	8 (19%)	5 (13%)	0.417
	Occlusion	5 (6%)	3 (7%)	2 (5%)	0.685
MCA	Stenosis	8 (10%)	3 (7%)	5 (13%)	0.414
	Occlusion	2 (2%)	2 (5%)	–	–
PCA	Occlusion	1 (1%)	1 (2%)	–	–
BA	Occlusion	1 (1%)	1 (2%)	–	–
VA	Stenosis	4 (5%)	3 (7%)	1 (3%)	0.329
	Occlusion	4 (5%)	4 (10%)	–	–
Several	Occlusions	5 (6%)	5 (12%)	–	–

*BA, basilar artery; ICA, internal carotid artery; MCA, middle cerebral artery; PCA, posterior cerebral artery; VA, vertebral artery*.

Comparing symptomatic vs. asymptomatic patients, no significant differences were found in univariable analysis in TMAO levels [3.96 (IQR 2.30–6.73) vs. 5.36 (3.59–8.68) μmol/L; *p* = 0.055], GFR [87 (72–97) vs. 82 (71–90) ml/min^*^1.73 m^2^; *p* = 0.189], and age [71 (60–79) vs. 69 (67–75) years; *p* = 0.756]. In multivariable analysis using binary logistic regression, TMAO was not a predictor of symptomatic cerebrovascular disease after adjusting for age, smoking, and GFR [OR 1.003 (95% CI: 0.941–1.070); *p* = 0.920]. However, smoking remained an independent discriminator between symptomatic and asymptomatic patients [OR 3.12 (1.13–8.66); *p* = 0.029; Table 3].

**Table 3:** Logistic regression analysis with symptomatic vs. asymptomatic cerebrovascular atherosclerosis as dependent variable.

**Parameter**	**OR**	**95% CI**	***p*-value**
Age	1.01	0.96–1.07	0.630
Smoking	3.12	1.13–8.66	0.029
GFR	1.02	0.99–1.05	0.288
TMAO	1.00	0.94–1.07	0.920

*CI, confidence interval; OR, odds ratio*.

As there were more patients with vessel occlusion in the symptomatic group, we performed a sensitivity analysis, excluding patients with occlusion of brain-supplying arteries (*n* = 63). In univariable analysis, there was again no statistically significant difference between symptomatic and asymptomatic patients in age [70 (62–80) vs. 69 (67–75); *p* = 0.522], GFR [83 (71–93) vs. 82 (70–90); *p* = 0.497], and TMAO [5.16 (3.12–8.58) vs. 5.91 (3.60–8.87); *p* = 0.496].

Moreover, there was no difference in the location of stenosis (*p* = 0.082). More data can be found in Table 4. Smoking showed a statistical trend that did not reach significance. In multivariable analysis, comparable to the primary analysis, TMAO was not a significant predictor of symptomatic stenosis while smoking was [TMAO: OR 1.039 (0.965–1.120), *p* = 0.311; smoking: OR 3.298 (1.035–10.495), *p* = 0.044].

**Table 4:** Baseline characteristics and TMAO values of the sensitivity analysis.

**Parameter *n* (%) or median (IQR)**	**Total**	**Symptomatic**	**Asymptomatic**	***p*-value**
Total number of patients	63	25	38	–
Age	69 (64–77)	70 (62–80)	69 (67–75)	0.522
Male gender	42 (67%)	19 (76%)	23 (61%)	0.202
Atrial fibrillation	7 (11%)	2 (8%)	15 (40%)	0.856
Hypertension	60 (95%)	25 (100%)	35 (92%)	0.150
Diabetes	19 (30%)	10 (40%)	9 (24%)	0.167
Coronary heart disease	13 (21%)	6 (24%)	7 (18%)	0.592
Peripheral artery disease	7 (11%)	1 (4%)	6 (16%)	0.145
Current smoker	19 (30%)	11 (44%)	8 (21%)	0.052
GFR (ml/min*1.73 m^2^)	82 (71–91)	83 (71–93)	82 (70–90)	0.497
TMAO (μmol/L)	5.73 (3.53–8.70)	5.16 (3.12–8.58)	5.91 (3.60–8.87)	0.496

TMAO did not show a correlation with the degree of carotid artery stenosis in symptomatic and asymptomatic patients (*n* = 52, Spearman-Rho coefficient 0.131; *p* = 0.354), nor was there a significant difference in univariable analysis (*p* = 0.162). Furthermore, there was no significant correlation of the NIHSS score on admission with TMAO values in symptomatic patients in univariable analysis (Spearman-Rho −0.022, *p* = 0.892).

## Discussion

In our study, we found that TMAO levels did not differ between symptomatic and asymptomatic participants with cerebrovascular atherosclerosis. Moreover, there was no difference between participants with symptomatic and asymptomatic stenosis of brain supplying vessels.

Our results are in line with a recent study (7), which included 296 patients (196 patients with ischemic stroke and 100 patients with a low cardiovascular risk profile). TMAO levels were significantly higher in stroke patients compared to controls, but there was no difference between patients with different stroke etiologies. Especially, there was no difference in TMAO levels between patients with cardioembolic and large-artery atherosclerosis etiology, which was expected from the data on TMAO and atherosclerosis (12). A possible explanation might be a comparable dysbiosis in patients with symptomatic and asymptomatic atherosclerosis, which was found by Yin et al. (13).

These results are supported by another study, showing that TMAO levels are also elevated in cardioembolic stroke due to atrial fibrillation (14). Liang et al. found that in 68 patients with atrial fibrillation and ischemic stroke, compared to 111 patients with atrial fibrillation without ischemic stroke, TMAO levels were significantly higher in patients with ischemic stroke (8.25 ± 1.58 vs. 2.22 ± 0.09 μmol/L, *p* < 0.01). However, these results were not adjusted to kidney function.

Another study found that TMAO levels are elevated in patients who develop new DWI lesions after CAS (4). In addition, the authors found that TMAO levels were significantly different between symptomatic and asymptomatic carotid artery stenosis [4.21 (2.35–5.86) vs. 3.24 (2.13–5.24) μmol/L, *p* = 0.012], seemingly contradicting our results. However, a valid comparison cannot be performed, as GFR values between the groups have not been reported and the results have not been adjusted for kidney function, which can be a major source of bias (1). Another difference to our study is that the patients were recruited in China, while our patients were recruited in Germany, potentially causing an ethnicity bias. Notably, our results showed higher TMAO levels compared to the study of Wu et al. (4) for both symptomatic and asymptomatic stenosis with slightly higher levels in asymptomatic cerebrovascular atherosclerosis. It is therefore unlikely that the association would be reversed if more patients would have been included.

While it is obvious that GFR has a strong influence on TMAO values (1), there is evidence that TMAO is a so-called gut-derived uremic toxin, which promotes atherosclerosis and is a therapeutic target despite being highly dependent on kidney function (15). Nevertheless, an adjustment for kidney function should be performed.

In the study of Bogiatzi et al. (15), TMAO was associated with carotid artery plaque burden, which could not be reproduced in our study. One reason might be the different selection criteria, as we only analyzed patients with hemodynamically relevant carotid artery stenosis (in the correlation analysis of degree of stenosis with TMAO), while Bogiatzi et al. included patients with any carotid artery plaque and measured the total plaque area, including a broader range of atherosclerosis.

Inconsistencies regarding the significance of TMAO levels even from larger studies have been presented before. While one large study showed that TMAO was a predictor of MACE in patients after acute coronary syndrome (2), another showed that there was no association to myocardial infarction but to strokes (16). With our study, the controversy on the significance of TMAO continues and the discussion on whether it can predict ischemic stroke in patients with asymptomatic stenosis has to be continued.

Our study has several limitations. While the TOAST criteria are accepted and validated, the inclusion of patients with vessel occlusions might create a distortion. However, our results were reproduced in the subset of patients with stenosis only. Moreover, the number of patients is relatively small as we set out to obtain the plasma sample of patients immediately on admission in the emergency room. Previous studies have shown that the timing of TMAO measurement is paramount (7, 17). We do not have any follow-up data on the patients with asymptomatic cerebrovascular atherosclerosis, so that we cannot answer the question, whether TMAO would predict future cerebrovascular events. There were some imbalances in the medication between the two groups. This is due to the design, as asymptomatic patients had known large-artery atherosclerosis that was treated with medication. However, we cannot exclude that this difference in medication might have caused a distortion of the results.

In summary, while TMAO levels have been proven to be elevated in patients with ischemic stroke, there are inconsistencies as they are elevated in asymptomatic cerebrovascular atherosclerosis in some studies but not in ours, and they are elevated in atherosclerosis but also in other stroke etiologies such as atrial fibrillation as well as its reported association with platelet hyperreactivity and the risk for thrombosis. It also remains unclear which patients would benefit from TMAO level measurement in clinical routine. Ultimately, it is yet to be determined whether influencing TMAO will be beneficial for patient outcome.

## Data Availability Statement

The datasets presented in this article are not readily available because of ethical restrictions. Requests to access the datasets should be directed to Sibu Mundiyanapurath, sibu.mundiyanapurath@med.uni-heidelberg.de.

## Ethics Statement

The studies involving human participants were reviewed and approved by Ethics committee of the Ruprecht-Karls-University Heidelberg (S-521/2017). The patients/participants provided their written informed consent to participate in this study.

## Author Contributions

MH, KD, AB, and MZ contributed to acquisition and cleaning of data, critical revision, and final approval of the manuscript. CS, PM, KS, JH, and JO contributed to study design, acquisition and cleaning of data, critical revision, and final approval of the manuscript. PR contributed to critical revision and final approval of the manuscript. SM contributed to study design, acquisition and cleaning of data, data analysis, data interpretation, manuscript drafting, critical revision, and final approval of the manuscript. All authors contributed to the article and approved the submitted version.

## Conflict of Interest

The authors declare that the research was conducted in the absence of any commercial or financial relationships that could be construed as a potential conflict of interest.
